# Immunotherapy in the Battle Against Bone Metastases: Mechanisms and Emerging Treatments

**DOI:** 10.3390/ph17121591

**Published:** 2024-11-26

**Authors:** Fatheia N. Hamza, Khalid Said Mohammad

**Affiliations:** 1 Department of Biochemistry, College of Medicine, Alfaisal University, Riyadh 11533, Saudi Arabia; fahamza@alfaisal.edu; 2 Department of Anatomy and Genetics, College of Medicine, Alfaisal University, Riyadh 11533, Saudi Arabia

**Keywords:** bone metastases, immunotherapy, tumor microenvironment, immune checkpoint inhibitors, osteoimmunology

## Abstract

Bone metastases are a prevalent complication in advanced cancers, particularly in breast, prostate, and lung cancers, and are associated with severe skeletal-related events (SREs), including fractures, spinal cord compression, and debilitating pain. Conventional bone-targeted treatments like bisphosphonates and RANKL inhibitors (denosumab) reduce osteoclast-mediated bone resorption but do not directly impact tumor progression within the bone. This review focuses on examining the growing potential of immunotherapy in targeting the unique challenges posed by bone metastases. Even though immune checkpoint inhibitors (ICIs) have significantly changed cancer treatment, their impact on bone metastases appears limited because of the bone microenvironment’s immunosuppressive traits, which include high levels of transforming growth factor-beta (TGFβ) and the immune-suppressing cells, such as regulatory T cells (Tregs) and myeloid-derived suppressor cells (MDSCs). This review underscores the investigation of combined therapeutic approaches that might ease these difficulties, such as the synergy of immune checkpoint inhibitors with agents aimed at bones (denosumab, bisphosphonates), chemotherapy, and radiotherapy, as well as the combination of immune checkpoint inhibitors with different immunotherapeutic methods, including CAR T-cell therapy. This review provides a comprehensive analysis of preclinical studies and clinical trials that show the synergistic potential of these combination approaches, which aim to both enhance immune responses and mitigate bone destruction. By offering an in-depth exploration of how these strategies can be tailored to the bone microenvironment, this review underscores the need for personalized treatment approaches. The findings emphasize the urgent need for further research into overcoming immune evasion in bone metastases, with the goal of improving patient survival and quality of life.

## 1. Introduction

Bone metastases, a condition where cancer cells spread from the primary tumor to the bones, are a prevalent and serious complication in various cancers, specifically breast, prostate, and lung cancers. This spread profoundly impacts patient prognosis and quality of life, often leading to severe skeletal-related events (SREs) such as pathological fractures, spinal cord compression, and debilitating pain. Effective therapies not only aim to ease pain but also to prevent SREs through various interventions, such as bisphosphonates and RANKL inhibitors like denosumab, which have shown promise in reducing osteoclast-mediated bone resorption [1,2]. The intricate and multifactorial nature of bone metastases makes it a challenging condition to manage, underscoring the need for effective therapeutic strategies. Over the past decades, immunotherapy has emerged as a transformative approach in oncology, with the potential to harness the immune system’s power to combat cancer. However, its efficacy in treating bone metastases remains limited because of the unique characteristics of the bone microenvironment.

The metastatic process leading to bone involvement is complex, involving multiple stages: invasion, circulation, extravasation, and colonization [3]. Initially, cancer cells from the primary tumor gain the ability to invade surrounding tissues through processes like epithelial-mesenchymal transition (EMT) and the degradation of the extracellular matrix (ECM) by matrix metalloproteinases (MMPs). This allows them to breach tissue barriers and enter the bloodstream (intravasation). Once in circulation, circulating tumor cells (CTCs) must survive immune surveillance and shear stress within the vascular system, a feat that only a small fraction of CTCs achieve. Those that survive eventually extravasate into the bone microenvironment, which is extremely favorable for tumor colonization because of its abundant supply of growth factors and cellular niches.

The bone microenvironment itself plays a critical role in fostering tumor growth. Cancer cells interact with bone-resident cells, such as osteoblasts and osteoclasts, disrupting the normal bone remodeling processes. This leads to either osteolytic or osteoblastic lesions, depending on the type of cancer. For instance, prostate cancer cells often promote bone formation (osteoblastic), while breast cancer and lung cancer cells are more likely to cause bone resorption (osteolytic). These interactions between tumor cells and bone cells create a vicious cycle: tumor cells release factors like parathyroid hormone-related protein (PTHrP) that stimulate osteoclasts to break down bone, releasing growth factors such as TGF-β that, in turn, promote tumor growth. This cycle of bone destruction and tumor proliferation makes bone metastases particularly difficult to treat.

A burgeoning field known as osteoimmunology has shed light on the role immune cells play in bone metabolism and metastasis. The dynamic interactions between immune cells (such as T cells, macrophages, and myeloid-derived suppressor cells) and bone cells (osteoclasts and osteoblasts) create a complex environment where immune responses are both modulated and manipulated by tumor cells. For example, T regulatory cells (Tregs) and myeloid-derived suppressor cells (MDSCs) contribute to immune suppression within the bone, allowing metastatic cancer cells to evade immune detection. In contrast, certain T cell subsets, like CD8+ cytotoxic T cells, have the potential to recognize and kill cancer cells, but the immunosuppressive microenvironment often blunts their efficacy.

Despite the promise of immunotherapy in many cancers, researchers have faced significant challenges in applying it to bone metastases. One of the primary obstacles is the immunosuppressive nature of the bone microenvironment. High levels of transforming growth factor-beta (TGFβ), a cytokine prevalent in bone metastases, suppress immune responses by inhibiting effector T cells and promoting the recruitment of immunosuppressive cells like Tregs. This dampened immune activity limits the effectiveness of immune checkpoint inhibitors (ICIs) in treating bone metastases, as these therapies rely on a functional immune response to be effective. Furthermore, the bone marrow niche itself provides a sanctuary for disseminated tumor cells, protecting them from immune surveillance and allowing them to remain dormant before reactivating and causing disease progression.

Current research is exploring various strategies to overcome these limitations, particularly through combination therapies. Combining ICIs with bone-targeted treatments like bisphosphonates or denosumab, which inhibit osteoclast-mediated bone resorption, has shown promise in enhancing therapeutic outcomes. Additionally, radiotherapy, known for its ability to target local bone metastases, may have immunomodulatory effects that can complement immunotherapy. The “abscopal effect”, where localized radiation induces a systemic anti-tumor immune response, has been observed in some cases, offering a potential avenue for enhancing immunotherapy’s effectiveness in bone metastases.

The development of more effective immunotherapy for bone metastases is critical, given the significant impact of bone metastases on patient outcomes. Although current treatments remain limited, ongoing research into the biology of bone metastases and immune system interactions offers hope for more targeted, personalized therapies. Future directions involve not only combining immunotherapy with existing therapies but also developing new strategies to manipulate the bone microenvironment in ways that enhance the immune system’s ability to combat cancer cells.

This review will explore the pathophysiology of bone metastases, with a focus on the interactions between the immune system and bone cells, the challenges faced in utilizing immunotherapy in this context, and emerging therapeutic strategies aimed at improving patient outcomes. By understanding these complex dynamics, we can work towards developing more effective treatments that target both the tumor cells and the bone microenvironment, offering hope for improved prognosis and quality of life for patients suffering from bone metastases.

## 2. Bone Metastases: An Overview

Bone metastases refer to the spread of cancer cells from a primary tumor to the bone, leading to secondary tumors within the skeletal system. This process is a significant complication in various cancers, particularly breast, prostate, and lung cancers, and is associated with considerable morbidity and mortality. The presence of bone metastases can significantly impact patient prognosis and quality of life, often leading to complications such as pain, fractures, and hypercalcemia [4,5]. The mechanisms underlying bone metastasis are complex and involve multiple stages, including invasion, intravasation, circulation, extravasation, and colonization. Understanding these stages is crucial for developing effective therapeutic strategies and improving patient outcomes [6].

### 2.1. The Metastatic Cascades

The first stage of bone metastasis is invasion, where cancer cells breach the surrounding tissue of the primary tumor. This process often involves the degradation of the extracellular matrix (ECM) and the basement membrane, facilitated by proteolytic enzymes such as matrix metalloproteinases (MMPs) [7,8]. Tumor cells undergo epithelial-mesenchymal transition (EMT), which enhances their migratory and invasive capabilities [9]. The tumor microenvironment plays a crucial role in promoting invasion by providing growth factors and signaling molecules that support cancer cell motility [10].

Following the invasion, cancer cells enter the bloodstream through a process known as intravasation. This step is critical as it allows tumor cells to disseminate throughout the body. The interaction between tumor cells and endothelial cells lining blood vessels is essential for successful intravasation. Tumor cells can exploit various mechanisms, such as the expression of adhesion molecules, to facilitate their entry into circulation [11]. For example, circulating tumor cells (CTCs) often express E-selectin ligands that promote their adhesion to the endothelium, enhancing their chances of intravasation [12].

Once in circulation, tumor cells face numerous challenges, including immune surveillance and mechanical shear stress. Only a small fraction of CTCs survive this hostile environment, with estimates suggesting that approximately 0.01% of CTCs are responsible for successful metastatic colonization [13]. The ability of tumor cells to evade immune detection is crucial for their survival in the bloodstream. Some cancer cells can express immune checkpoint proteins that inhibit T-cell activation, allowing them to persist in circulation. Furthermore, the presence of specific chemokines and growth factors in the blood can aid in the survival of CTCs, providing them with the necessary signals to thrive [7].

The next stage is extravasation, where circulating tumor cells exit the bloodstream and invade the surrounding tissues of the bone. The interaction between tumor cells and the endothelial cells of the bone vasculature facilitates this process. Tumor cells can adhere to the endothelium through various adhesion molecules, allowing them to penetrate the vessel wall and enter the bone microenvironment [14]. The bone marrow niche is particularly favorable for metastasis due to its unique microenvironment, which supports tumor cell survival and growth [15]. Factors such as the presence of hematopoietic stem cells and the rich supply of growth factors in the bone marrow contribute to the successful extravasation of tumor cells [16].

Once tumor cells have successfully extravasated into the bone, they enter the colonization phase. This stage involves the establishment of a metastatic tumor within the bone microenvironment. Tumor cells can remain dormant for extended periods before reactivating and proliferating, which complicates treatment strategies [10]. The interaction between tumor cells and the bone stroma is critical for successful colonization. Tumor cells can secrete factors that alter the bone microenvironment, promoting their own growth while disrupting normal bone remodeling processes [17]. For instance, the release of osteoclast-activating factors leads to increased bone resorption, creating a favorable niche for tumor growth [18]. Additionally, the expression of specific genes associated with osteotropism, such as SFRP2, can enhance the ability of cancer cells to adapt to the bone environment [18].

### 2.2. The Bone Microenvironment

Bone is fundamentally distinguished by its inherently dynamic and perpetually evolving characteristics, which serve to define it as a specialized form of tissue that actively participates in a complicated and intricate process of remodeling; this complex phenomenon occurs as a direct consequence of a diverse array of physical activities and varying metabolic demands that manifest throughout the individual’s lifespan and influence the structural and functional integrity of the skeletal system. The osteoblast, which plays a pivotal role in the synthesis and formation of bone matrix, alongside the osteoclast, which is primarily responsible for the resorption and breakdown of bone tissue, serves as the principal cellular players that contribute to both the intricate process of bone remodeling and the pathological development of metastatic lesions within the skeletal system [19,20,21]. When malignant tumor cells invade and infiltrate the bony matrix, they disrupt the established and normal processes of bone remodeling that are crucial for maintaining skeletal integrity. Specifically regarding prostate cancer, neoplastic cells seem to aid and promote bone formation, whereas, in instances of breast, lung, and renal cancers, there is a clear increase in bone resorption, showcasing the varied influences of different cancers on bone metabolism [22,23]. The osteoclast’s main role in breaking down bone tissue, which is essential for understanding the development of bone metastases, has gained significant attention in research over the past few decades, shedding light on the processes involved in cancer advancement. The development of therapeutic agents that specifically target the osteoclast and their current use in the management and treatment of bone metastases, as well as various metabolic bone disorders, proves the clinical relevance of these cellular processes in oncology [24,25]. While osteoblasts and osteoclasts have traditionally been recognized as the primary cellular actors involved in the complex formation of bone metastases, emerging scientific investigations have demonstrated that other cell types residing within the bone marrow milieu also play a significant and influential role in the trajectory of disease progression, thereby expanding our understanding of the interactions within the bone microenvironment [26].

The interactions between cancer cells and the bone microenvironment influence the complex biology of bone metastasis. The “seed and soil” hypothesis, proposed by Paget, suggests that certain cancer cells, the “seeds”, have a predilection for specific organs, the “soil”, that provide a conducive environment for their growth [27]. In the case of bone metastases, the bone microenvironment is rich in growth factors, cytokines, and extracellular matrix components that can promote tumor cell survival and proliferation [27,28]. For instance, osteoblasts and osteoclasts in the bone facilitate a unique milieu that can either support or inhibit tumor growth, depending on the type of cancer and its interaction with these cells [29].

The distribution of bone metastases varies by the type of cancer and has significant clinical implications. Breast cancer metastases are more frequently found in the axial skeleton, including the spine and ribs, while prostate cancer metastases often localize to the vertebrae and pelvis [30]. This distribution reflects the underlying biological mechanisms that govern tumor cell homing and colonization in the bone. The presence of skeletal-related events (SREs), such as pathological fractures and spinal cord compression, profoundly affects the quality of life and overall survival of patients with bone metastases [31,32].

Several factors influence the prognosis for patients with bone metastases, including the type of primary cancer the involvement of skeletal, and other visceral metastases. Individuals diagnosed with breast and prostate cancers, particularly those who have developed bone metastases, tend to exhibit a more favorable prognosis when evaluated against the cohort of patients whose malignancies have metastasized to the lung tissue. This shows a significant variance in outcomes based on the specific type of metastatic spread involved in these distinct forms of cancer [33,34]. This difference is partly due to the biological behavior of the tumors and their interaction with the bone microenvironment, which can either promote or inhibit metastatic growth [33].

## 3. The Immune System Interplay in Bone Metastases

### 3.1. Overview of the Immune System in Cancer

The immune system plays a crucial role in recognizing and eliminating cancer cells through a dynamic interplay between innate and adaptive immune responses. Cancer cells often express antigens that host T cells can recognize, but successful tumors have developed strategies to evade these immune defenses [35].

The innate and adaptive components of the immune system collectively engage in a highly significant and complex process aimed at the eradication of malignant cancer cells, a phenomenon that is scientifically referred to as the Cancer-Immunity Cycle [36]. This detailed sequence includes various important phases: At the outset, tumor-derived antigens are absorbed by specialized immune cells referred to as dendritic cells (DCs), which not only manage these antigens but also produce an array of pro-inflammatory cytokines and numerous immunological elements that greatly influence the immune response. In the next step, dendritic cells take the processed tumor antigens and display them to T cells, prompting the next phase that includes the priming and further stimulation of effector T cells, consequently launching a chain reaction focused on tackling tumor development. When T cells are activated, they subsequently infiltrate the tumor microenvironment, enabling them to discern and attach to cancer cells through a uniquely specific interaction that involves their T-cell receptors (TCR) [37]. Ultimately, this dynamic interaction culminates in the targeted destruction of the neoplastic cells, which is achieved through the actions of activated cytotoxic T cells that are tailored to eliminate such threats to the organism’s health.

### 3.2. Innate and Adaptive Immunity

Innate immune cells contribute to the activation of adaptive immune cells. A famous example is the Antigen Presenting Cells (APCs), such as macrophages, which activate the tumor-killing activity of T lymphocytes [38]. Innate immunity comprises different types of cells with pattern recognition receptors (PPRs). Innate immune cells play complex roles in tumor biology, with some subsets capable of detecting and eliminating tumors while others can facilitate tumor growth and metastasis. Macrophages, for example, can exhibit either anti-tumor (M1-like) or pro-tumor (M2-like) functions depending on their polarization state and the tumor microenvironment. Similarly, neutrophils can act as anti-tumor (N1-like) cells that inhibit tumor growth or as pro-tumor (N2-like) cells that promote tumor proliferation and invasion. Understanding the factors that dictate these divergent functions is critical for developing therapeutic strategies targeting innate immunity in cancer. Innate immune cells, including natural killer (NK) cells and macrophages, are first responders in the tumor microenvironment. They can recognize and exterminate cancer cells by directly detecting abnormal features, such as the expression of stress-induced molecules. These cells also produce cytokines that can modulate the tumor microenvironment and influence the recruitment and activation of adaptive immune cells [39].

Adaptive immunity, particularly involving T cells, plays a pivotal role in the immune response to cancer. CD8+ T cells can recognize and destroy cancer cells that present specific antigens on their surface. The effectiveness of these T cells can be significantly influenced by the presence of helper T cells (CD4+ T cells), which enhance the cytotoxic function of CD8+ cells and help in the maintenance of a long-term immune response [40].

Although the ideal situation is that the immune system recognizes and eliminates the transformed cells, tumors develop various immune evasion strategies [39]. One common tactic is the creation of a highly suppressive tumor microenvironment that inhibits effective immune surveillance and killing. Tumors may recruit regulatory T cells (Tregs) and myeloid-derived suppressor cells (MDSCs), which suppress the function of effector T cells [41,42]. Many tumors express checkpoint proteins like PD-L1, which bind to inhibitory receptors on T cells (e.g., PD-1), leading to T cell exhaustion.

The current advancements in immunotherapy, such as checkpoint inhibitors that block PD-1/PD-L1 interactions and therapies that modulate the tumor microenvironment to enhance immune activity, are based on these interactions. Understanding and manipulating the complex interplay between cancer and the immune system has become a focal point in developing effective cancer treatments, aiming to reinstate the immune system’s ability to fight cancer.

### 3.3. Immune Influence on Bone Metabolism

The emerging field of osteoimmunology explores how immune cells influence bone remodeling and homeostasis, encapsulating this relationship. Bone remodeling is a continuous process involving the coordinated actions of osteoclasts, which resorb bone, and osteoblasts, which form new bone. This balance is crucial for maintaining skeletal integrity and mineral homeostasis, and immune cells play a pivotal role in regulating these processes. Furthermore, the bone marrow serves as a critical niche for hematopoiesis and the differentiation of immune cells. The microenvironment of the bone marrow is rich in signaling molecules that facilitate the development and function of both osteoblasts and immune cells [43,44]. This interplay is vital for the maintenance of bone integrity and the regulation of immune responses, specifically in the context of bone remodeling and repair following injury [45,46].

Studies have shown that immune cells, especially T cells, B cells, and myeloid-derived cells, play a significant role in influencing bone metabolism in a study that looked at the role of T regulatory cells (Tregs) in osteogenesis imperfecta (OI), OI mice had a reduced number of Tregs, which led to increased activation of T cells and higher levels of pro-inflammatory cytokines, such as IFN-g and TNF-a. This pro-inflammatory environment exacerbated bone loss by increasing osteoclast activity, cells responsible for bone resorption. Transplanting Tregs into OI mice was able to reduce inflammation, decrease osteoclast numbers, and enhance bone formation by increasing osteoblast mineralization. These changes significantly improved bone remodeling, leading to stronger bones with improved mechanical properties [47]. Conversely, regulatory T cells (Tregs) can exert protective effects against excessive bone loss by inhibiting osteoclast formation through the production of anti-inflammatory cytokines. Osteoclasts can induce the formation of a specific type of regulatory T-cells (Foxp3+ CD8 T-cells), which play a protective role in limiting bone resorption [48]. It was found mice lacking CD8 T-cells experienced greater bone loss, while those treated with Foxp3+ CD8 T-cells showed reduced bone loss and increased bone density. These regulatory T-cells were shown to not only reduce the number of osteoclasts but also decrease the activity of pro-inflammatory effector T-cells, promoting bone formation [48,49].

The role of B cells in bone metabolism is also noteworthy. They can produce antibodies and cytokines that influence osteoclast and osteoblast function. For example, B cells can secrete osteoprotegerin (OPG), a decoy receptor that inhibits RANKL (receptor activator of nuclear factor kappa-Β ligand) signaling, thereby reducing osteoclastogenesis [50]. In a mouse model of bone loss caused by ovariectomy, a model for postmenopausal osteoporosis, it was shown that B lymphocytes, not T lymphocytes, contribute significantly to bone loss through RANKL expression. Using genetically modified mice that lack RANKL expression, specifically in B cells, it was found that these mice were partially protected from bone loss after ovariectomy, particularly in trabecular bone. This protection was linked to a failure to increase the number of osteoclasts in the absence of RANKL. Deletion of RANKL in B cells did not affect bone mass in healthy mice, indicating that B cell RANKL plays a role predominantly in the context of estrogen deficiency [51].

Myeloid-derived suppressor cells (MDSCs) are another class of immune cells that have been implicated in bone metabolism. These cells can regulate osteoclastogenesis and bone resorption through the production of various cytokines and signaling molecules [52,53]. The presence of MDSCs in the bone marrow microenvironment suggests a complex regulatory network where these cells can modulate the activity of both osteoclasts and osteoblasts, thereby influencing overall bone remodeling [52,54]. Recent studies have emphasized the significance of MDSCs as a potential marker for bone healing. Distinct systemic immune profiles, especially elevated levels of myeloid-derived suppressor cells (MDSCs) and interleukin-10 (IL-10) correlated inversely with bone healing. These immune markers were predictive of poor healing outcomes, even as early as one week after treatment [55].

It was observed that mice deficient in CD80/86 exhibited osteopenia due to increased osteoclast differentiation. This was linked to a failure in the usual inhibition mechanism of osteoclast formation by regulatory T cells and CTLA-4. Mechanistically, the engagement of CD80/86 by CTLA-4 activates the enzyme indoleamine 2,3-dioxygenase (IDO) in osteoclast precursors, which breaks down tryptophan and promotes apoptosis in these cells. Additionally, IDO-deficient mice also displayed increased osteoclast numbers and bone loss. These findings were validated in human models, where therapeutic interventions targeting the CD80/86-CTLA-4 interaction influenced osteoclast precursor numbers and osteoclastogenesis [56]. In contrast, anti-inflammatory cytokines, such as IL-10 and transforming growth factor-beta (TGFβ), can promote osteoblast activity and inhibit osteoclastogenesis, highlighting the dual role of immune cells in bone metabolism [50,57,58].

The concept of “osteoimmunology” emphasizes the bidirectional communication between bone and immune systems. For instance, bone cells can influence immune cell function through the secretion of signaling molecules that modulate the immune response. Osteoblasts and osteocytes express various receptors and ligands that interact with immune cells, thereby shaping the immune landscape within the bone [59,60]. This crosstalk is essential for maintaining bone homeostasis and responding to pathological conditions such as osteoporosis and inflammatory bone diseases [61]. More recent findings challenged the traditional understanding of bone homeostasis. Using germ-free (GF) and specific-pathogen-free (SPF) mice, it was found that commensal gut microbiota exerts both anti-anabolic and pro-catabolic effects on skeletal remodeling, leading to reduced bone formation and increased bone resorption in healthy adults. In SPF mice, the microbiota suppresses osteoblast and promotes osteoclastogenesis, resulting in bone loss. The study also introduces the concept of a “Gut-Liver-Bone Axis”, suggesting that the liver plays a role in mediating these effects via immune responses driven by the gut microbiota. Elevated pro-inflammatory markers in the liver and bone marrow were linked to enhanced osteoclast maturation and bone erosion [62].

### 3.4. Immune System and Bone Metastases

The immune system assumes a complex function in the ontogeny and advancement of bone metastases, markedly affecting the tumor microenvironment and the dynamics between neoplastic cells and immunological cells. Bone metastases are a common complication in various cancers, including breast, prostate, and lung cancers, and they often lead to debilitating skeletal-related events. Understanding the immune system’s involvement in this process is crucial for developing effective therapeutic strategies.

Bone metastases create a unique microenvironment characterized by a complex interplay between tumor cells, bone cells, and immune cells. This microenvironment is often immunosuppressive, allowing tumor cells to evade immune surveillance. The “bone marrow niche” is crucial, as it provides a protective environment for disseminated tumor cells, shielding them from immune detection and response [63]. The local immune landscape in bone metastases is often skewed towards an immunosuppressive phenotype, with an increased presence of regulatory T cells (Tregs) and myeloid-derived suppressor cells (MDSCs), which further inhibit effective anti-tumor immunity [64,65].

**Figure 1:**
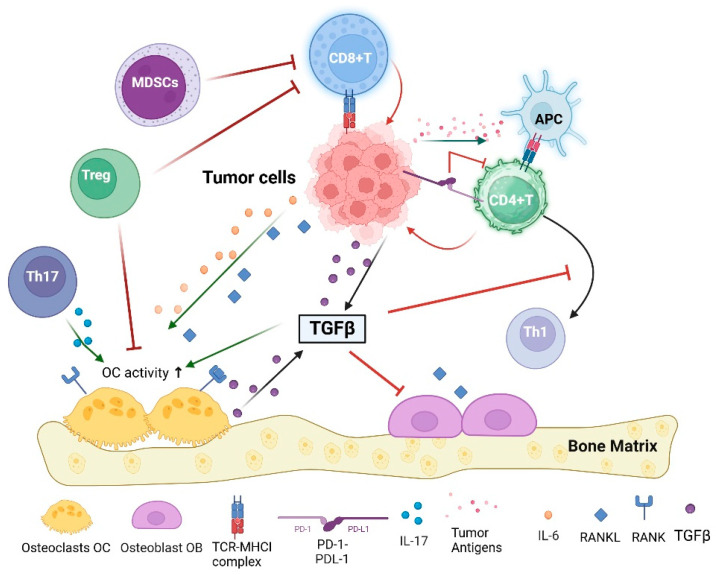
Interplay of Immune Responses and Bone Microenvironment in Cancer Metastasis. This figure illustrates the interactions between the immune system and bone microenvironment during cancer metastasis. Central to this process are the tumor cells, which express specific antigens recognized by CD8+ T cells via the TCR-MHCI complex. These interactions are modulated by immune checkpoints, specifically the PD-1/PD-L1 pathway, highlighting the suppressive role of T cell activity. Various immune cells, including APCs, CD4+ T cells, regulatory T cells (Tregs), myeloid-derived suppressor cells (MDSCs), Th1, and Th17 cells, contribute to the dynamic immune landscape. Key cytokines such as TGFβ, IL-17, and IL-6, along with RANKL (secreted by cancer cells and osteoblasts), are pivotal in enhancing osteoclast (OC) activity promoting bone resorption. This figure underscores the dual role of the immune system in both supporting and combating bone metastasis, delineating targets for potential therapeutic interventions in oncology (Figure was created with BioRender.com).

The receptor activator of the nuclear factor-kappa B (RANK)/RANK ligand (RANKL) signaling pathway is a critical mediator in the relationship between the immune system and bone metastases (Figure 1). RANKL is produced by osteoblasts and is essential for osteoclast differentiation and activation, leading to bone resorption. The RANK/RANKL signaling axis not only facilitates osteoclastogenesis but also plays a pivotal role in modulating immune responses within the tumor microenvironment. Recent studies have indicated that RANKL can influence T-cell activation and dendritic cell survival, linking bone metabolism directly to immune regulation during cancer progression [66,67]. Tumor cells can exploit this pathway to promote their own survival and proliferation within the bone microenvironment [68,69]. Furthermore, RANKL not only facilitates bone remodeling but also influences immune responses, as it can modulate the activity of various immune cell types, including T cells and dendritic cells [68,70].

The vicious cycle of bone metastasis involves the interaction between tumor cells and the bone microenvironment, which is strongly influenced by immune factors. Tumor cells can induce osteoclastogenesis through the secretion of factors such as RANKL and macrophage colony-stimulating factor (M-CSF), leading to increased bone resorption and the release of growth factors that further support tumor growth [64,71]. Immune cells that promote tumor survival and growth exacerbate this cycle, creating an environment conducive to metastasis [72].

Immune checkpoint inhibitors (ICIs) have emerged as a promising therapeutic strategy for various cancers, including those with bone metastases. However, the efficacy of ICIs in this context is often limited by the immunosuppressive nature of the bone microenvironment. Studies have shown that the presence of bone metastases can diminish the effectiveness of ICIs, as local immune cells may not mount an effective anti-tumor response [65,73]. This emphasizes the need for combination therapies that can strengthen immune responses in the bone microenvironment, utilizing radiotherapy or other immunomodulatory agents [74,75].

The role of cytokines in the development of bone metastases is also significant. Transforming growth factor-beta (TGFβ) is a key cytokine that promotes tumor cell invasiveness and angiogenesis while also suppressing immune responses, allowing cancer cells to escape immune detection [76,77]. TGFβ signaling is often upregulated in bone metastases, contributing to the establishment of an immunosuppressive niche that facilitates tumor growth and progression [78]. Targeting TGF-β signaling pathways may represent a viable therapeutic strategy to disrupt this cycle and enhance anti-tumor immunity in patients with bone metastases [76,79].

Besides TGFβ, other cytokines and chemokines play crucial roles in modulating the immune response within the bone microenvironment. For instance, interleukin-6 (IL-6) and IL-8 have been implicated in promoting osteoclastogenesis and enhancing tumor cell survival [80]. The presence of these cytokines can lead to a further skewing of the immune landscape towards an immunosuppressive state, thereby facilitating the establishment and growth of bone metastases [80,81].

Tumor-associated macrophages (TAMs) can adopt different phenotypes that either promote or inhibit tumor growth. These phenotypic shifts in TAMs are influenced by various factors within the tumor microenvironment, such as cytokines and extracellular matrix components. The presence of interleukin-6 (IL-6) can skew TAMs towards a pro-tumorigenic M2 phenotype, enhancing their capacity to support cancer cell survival and metastasis [82]. Additionally, the intricate crosstalk between TAMs and other immune cells, like T lymphocytes, further complicates this dynamic; while some TAMs may suppress adaptive immunity, others could potentially enhance anti-tumor responses under certain conditions [83].

The immune system’s role in bone metastasis is not limited to promoting tumor growth; it can also contribute to the destruction of bone tissue. The interaction between immune cells and osteoclasts can lead to increased bone resorption, which is a hallmark of osteolytic metastases. This process is mediated by various factors, including RANKL and pro-inflammatory cytokines, which can exacerbate bone degradation and further support tumor growth [64,71]. The presence of pro-inflammatory cytokines such as interleukin-8 (IL-8) further exacerbates this destructive cycle by enhancing osteoclastogenesis and promoting bone resorption. Some studies have indicated that IL-8 not only stimulates osteoclast activity but also regulates RANKL expression in osteoblastic cells, thus linking inflammatory responses to metastatic bone disease more directly than previously understood [84]. This dual role of IL-8 highlights its potential as both a biomarker for aggressive cancer behavior and a therapeutic target, suggesting that modulating IL-8 levels could mitigate the adverse effects of bone metastases on patient morbidity [70].

## 4. Concept of Cancer Immunoediting

The concept of cancer immunoediting theory was introduced back in 2002 [85,86]. The role of immune cells in cancer immunoediting is pivotal in determining the progression or regression of tumors. Cancer immunoediting is a dynamic process that involves the immune system’s dual role in both suppressing and promoting tumor development. This theory suggests that the immune system can promote or inhibit the formation of tumors. This process includes three phases: elimination, equilibrium, and escape. During the elimination phase, which is the initial phase, both innate and adaptive immune systems identify and eliminate the tumor cells before clinical detection. Immune cells such as T-cells and natural killer cells identify and destroy nascent tumor cells [87]. This phase is crucial for cancer immunosurveillance, where the immune system actively suppresses tumor growth. If some tumor cells survive the elimination phase, they enter the equilibrium phase. The immune system and tumor cells reach a balance where tumor cells are not completely eradicated but are kept in check. This phase can lead to cancer dormancy, where tumor cells persist in a non-proliferative state, potentially contributing to therapy resistance and relapse [87]. Tumor cells that survive the equilibrium phase may acquire mutations that allow them to evade immune detection, leading to tumor progression [88,89]. It was demonstrated that, even in the absence of adaptive immune cells, the innate immune system—particularly through NK cells producing interferon-gamma (IFN-γ) can induce M1 macrophages to perform immunoediting. This leads to the suppression of tumor growth and increases the immunogenicity of tumor cells. The findings suggest that NK cells and M1 macrophages are critical effectors in cancer immunoediting, highlighting the importance of innate immune cells in controlling cancer development, even without adaptive immunity [90]. Immunoediting occurs not only during natural immune responses but also in response to immunotherapy, such as immune checkpoint inhibitors. Tumors can evolve to escape immune control through mechanisms like neoantigen loss, defects in antigen presentation, or the upregulation of immune checkpoint molecules [91].

## 5. Introduction to Immunotherapy

Cancer immunotherapy relies on two mechanisms of action: passive immunotherapy via anti-tumor antibodies or adoptive transfer of cytotoxic T and NK cells, and active immunotherapy, the activates the patient’s immune cells via checkpoints blockade. T cells can control cancer. Therefore, it is considered a drug, and it could be triggered in three major ways: by checkpoint inhibitors, adoptive transfer of genetically engineered T cells, and through induction in vivo by vaccination. In addition to these established methods, researchers are increasingly exploring the potential of combining various immunotherapeutic approaches to enhance overall efficacy. For instance, recent studies suggest that integrating checkpoint inhibitors with adoptive cell transfer therapies can yield synergistic effects, leading to improved patient outcomes in cancers such as melanoma and lung cancer. Advancements in understanding tumor microenvironments have revealed how modifying inflammatory signals could further optimize T-cell responses, thereby addressing challenges related to immune evasion by tumors. This multifaceted strategy not only aims to boost the activation and persistence of therapeutic T cells but also seeks to reshape the surrounding environment to support a more robust anti-tumor immunity, highlighting an exciting frontier in cancer treatment. As researchers continue to explore these innovative approaches, the potential for personalized medicine becomes increasingly apparent, allowing for tailored therapies that consider individual patient profiles and tumor characteristics.

### 5.1. Types of Immunotherapy

#### 5.1.1. Checkpoints Inhibitors (ICIs)

Immune checkpoint inhibitors have become the most widely used agents in cancer immunotherapy through disturbing the immune checkpoint pathway. In the tumor microenvironment, the immune checkpoints are upregulated, which hinders T-cell activation. For example, hypoxia, a common feature of the TME, significantly upregulates immune checkpoints, such as PD-1, PD-L1, and CTLA-4. This is mediated through pathways involving hypoxia-inducible factor-1α (HIF-1α), which regulates the expression of these checkpoints, thereby promoting immune evasion and tumor survival [92]. Targeting ICIs can unleash the power of T cells by eliminating the negative signals that block T-cell function. Cytotoxic T-lymphocyte-associated protein 4 (CTLA-4) is expressed on T cells and competes with the costimulatory receptor CD28 for binding to CD80/CD86 on antigen-presenting cells. This competition inhibits T cell activation and proliferation, thus serving as a brake on the immune response [93]. Tregs constitutively express CTLA-4, which is pivotal in maintaining immune homeostasis and preventing autoimmunity. Tregs utilize CTLA-4 to suppress effector T cell activity in the tumor microenvironment, facilitating tumor immune escape [94]. PD1 binds to programmed death ligand 1 (PD-L1) that is expressed by various immune cells and other neoplastic cells. Upon binding to its ligands PD-L1 or PD-L2, it delivers inhibitory signals that reduce T cell proliferation and cytokine production [95,96]. Combination therapies targeting these checkpoints, CTLA-4 and PD-1, increase progression-free survival in patients with metastatic melanoma [97]. However, in other types of cancer, these responses are uncommon.

#### 5.1.2. CAR T-Cell Therapy

Another promising cancer immunotherapy is the chimeric antigen receptor (CAR-T) cell. It represents a significant advancement in cancer treatment, particularly for hematologic malignancies. This innovative approach involves genetically modifying a patient’s T cells to express CARs, enabling them to recognize and destroy cancer cells. This therapy involves extracting T cells from a patient, genetically engineering them to express CARs, and reinfusing them into the patient. These CARs are designed to target specific antigens on cancer cells, allowing the modified T cells to identify and kill these cells [98]. CAR innate immune cells and cytokine therapy are examples of immunotherapy based on innate immune cells [38]. Clinical approval has been granted to several products that utilize CAR-T cell therapy and are now available in the market. The first CAR-T cell therapy to gain FDA approval was Kymriah, which was sanctioned in 2017 for the treatment of pediatric and young adult patients with R/R acute lymphoblastic leukemia (ALL). This was followed by Yescarta, approved for adult patients with R/R large B-cell lymphoma (DLBCL) [99,100]. The approval of these therapies marked a significant milestone in the field of oncology, as they provided new hope for patients who had limited treatment options available. The mechanism of action for these therapies involves the genetic modification of a patient’s T cells to express CARs that specifically target tumor-associated antigens, leading to the destruction of malignant cells [101,102]. DC-targeted adoptive cell transfer has also been approved for prostatic cancer [103]. Most clinically approved immunotherapy is T-cell centered. However, more attention needs to be considered to the innate immunity arm as its essential role is the capturing of the cancer antigen and activation of the adaptive immunity [41].

While its success in blood cancers is well-documented, the application of CAR-T cell therapy in solid tumors remains a challenge. Solid tumors often present a more complex microenvironment that shields the cancer cells from immune attack. Researchers are actively investigating ways to improve CAR-T therapy’s efficacy against solid tumors, including the identification of suitable targets and overcoming the tumor microenvironment’s immunosuppressive properties.

CAR-T cell therapy offers several advantages over traditional cancer treatments. Unlike chemotherapy and radiation, which can harm healthy cells, CAR-T therapy is highly specific, targeting only cancerous cells [104]. This specificity reduces the risk of collateral damage to normal tissues. Additionally, CAR-T cells have the potential to persist and proliferate in the body, offering long-term protection against cancer recurrence. However, CAR-T therapy is not without its limitations. One of the most significant challenges is cytokine release syndrome (CRS), a potentially life-threatening immune reaction caused by the rapid activation and expansion of CAR-T cells [105]. Other side effects include neurological toxicities, and the therapy’s high cost and complex production process remain barriers to widespread use [106].

#### 5.1.3. Cytokines and Other Immunomodulators

Cytokines such as GM-CSF can activate NK cells and promote the maturation of APC, which stimulates immunity and mediates the anti-tumor effects. Injections of GM-CSF in patients with melanoma have shown an increase in the number of tumor-infiltrating DCs [38]. This increase in tumor-infiltrating dendritic cells (DCs) is crucial, as these APCs play a key role in bridging innate and adaptive immunity by processing and presenting antigens to T cells. Furthermore, the combination of GM-CSF with other immunotherapeutic strategies, such as immune checkpoint inhibitors, has shown promise in enhancing anti-tumor responses. For instance, recent studies indicate that genetically engineered myeloid cells derived from induced pluripotent stem cells can significantly boost CD8+ T cell proliferation and promote effector cell infiltration within tumors, thereby amplifying the overall therapeutic efficacy [107].

#### 5.1.4. Cancer Vaccines

Vaccines are composed of antigens and adjuvants. The immune response involves DCs to these antigens that capture and present the antigen, which facilitates lymphocyte activation and subsequent immunity. One of the promising immunotherapy approaches is the cancer vaccine that uses dendritic cells. The administration of cancer antigen that contains synthetic peptides, antigen-expressing viruses, or tumor lysate could be captured and presented by endogenous DCs [103]. Several clinical trials are ongoing regarding DC-based vaccines [108].

#### 5.1.5. Newly Approved Drugs

Tecelra, also known as IT-139, is an innovative small molecule compound used in cancer treatment, particularly effective against tumors resistant to standard therapies. The safety and efficacy of afamitresgene autoleucel (afami-cel), an autologous T cell therapy designed to target melanoma-associated antigen A4 (MAGE-A4) in patients with solid tumors. Conducted as a multicenter Phase 1 trial, the study enrolled patients with relapsed/refractory metastatic cancers expressing MAGE-A4, including synovial sarcoma (SS). Results indicated that the therapy exhibited promising anti-tumor activity, particularly in SS patients, where the objective response rate was 44%, compared to 24% across all tumor types. Afami-cel demonstrated a robust safety profile with expected toxicities, including hematologic issues and cytokine release syndrome, which were manageable. Mechanistic insights revealed that afami-cel infiltrates tumors, trigger interferon-γ-driven responses, and activates adaptive immunity [109]. A phase 2 clinical trial study (SPEARHEAD-1) evaluated afamitresgene autoleucel (afami-cel), a T-cell therapy targeting MAGE-A4, in patients with advanced synovial sarcoma or myxoid round cell liposarcoma. The study involved heavily pre-treated patients across 23 sites. With a single dose of afami-cel after lymphodepletion, the therapy achieved a 37% overall response rate, including 39% in synovial sarcoma patients and 25% in those with myxoid round cell liposarcoma. While common adverse events included manageable cytopenias and cytokine release syndrome, no treatment-related deaths occurred. These findings highlight afami-cel’s potential as an effective T-cell receptor therapy for solid tumors, especially in challenging cases of advanced synovial sarcoma [110]. In the month of August in the year 2024, afamitresgene autoleucel (TECELRA^®^) received official approval from the regulatory authorities in the United States of America, granted under the expedited pathway commonly referred to as accelerated approval, specifically designated for the therapeutic intervention aimed at adult patients who are afflicted with unresectable or metastatic synovial sarcoma, provided that these individuals have previously undergone chemotherapy, possess the HLA-A*02:01P, -A*02:02P, -A*02:03P, or -A*02:06P positive genetic markers, and have tumors that exhibit the expression of the MAGE-A4 antigen, which must be verified through the use of companion diagnostic devices that have been either approved or cleared by the Food and Drug Administration (FDA) [111].

## 6. Tumor Immunogenicity

Tumor immunogenicity is a critical factor in determining the efficacy of cancer therapies, particularly immunotherapies. Tumors are often classified into “hot” and “cold” categories based on their immune microenvironment characteristics. “Hot tumors” are characterized by a high degree of immune cell infiltration, particularly T cells, and a robust immune response, making them more responsive to immunotherapy. In contrast, “cold tumors” exhibit low immune cell infiltration and a lack of effective immune response, which often leads to poor therapeutic outcomes [112].

Several factors can influence the classification of tumors as “hot” or “cold”, including the tumor microenvironment (TME) and the intrinsic properties of the tumor cells. Hot tumors typically have elevated levels of pro-inflammatory cytokines and immune cell types such as CD8+ T cells, which are essential for effective anti-tumor immunity [113]. For instance, melanoma and certain lung cancers are often categorized as hot tumors due to their significant T-cell infiltration and favorable immune contexture [113]. Conversely, cold tumors, such as triple-negative breast cancer (TNBC) and pancreatic ductal adenocarcinoma (PDAC), often have a desmoplastic stroma that limits immune cell access and function, resulting in a suppressed immune response [114].

Strategies to convert cold tumors into hot tumors to enhance the efficacy of immunotherapies have been an area of research focus in recent years. This transformation often involves inducing immunogenic cell death (ICD) in tumor cells, which can promote the release of tumor-associated antigens (TAAs) and enhance immune recognition [115]. Therapies that stimulate the immune system or alter the TME can lead to increased T-cell infiltration and activation, thereby converting a cold tumor into a hot one [116]. Additionally, the use of agents such as Z-100, derived from Mycobacterium tuberculosis, has been shown to enhance cytokine production and promote a hot tumor phenotype [117]. The dynamic nature of the TME also plays a crucial role in the immunogenicity of tumors. Factors such as hypoxia, metabolic changes, and the presence of immunosuppressive cells (e.g., myeloid-derived suppressor cells) can contribute to the cold phenotype of tumors [118]. Understanding these mechanisms is essential for developing effective therapeutic strategies aimed at reprogramming the TME to favor an immune-active state (reviewed by Xu et al. [119]).

The distinction between hot and cold tumors is pivotal in cancer immunology, influencing treatment strategies and patient outcomes.

## 7. Immunotherapy and Bone Metastases

### 7.1. Rationale for Immunotherapy in Bone Metastases

Immunotherapy has emerged as a promising therapeutic approach for a diverse array of malignancies; nevertheless, its effectiveness in individuals presenting with bone metastases remains constrained. A multitude of factors contribute to this predicament, mainly involving the distinct immunological and microenvironmental features of bone metastases that impede the efficacy of immunotherapeutic strategies. The bone microenvironment contains heightened concentrations of transforming growth factor-beta (TGFβ), which is a prominent obstacle that inhibits adaptive immune responses. Research has elucidated that the secretion of TGFβ in bone metastases results in a diminished population of T helper 1 (Th1) effector cells, consequently fostering resistance to immunotherapy [120,121]. This immunosuppressive environment is further exacerbated by regulatory T cells (Tregs) in the bone marrow, which can inhibit immune cell function and contribute to poor responses to treatment [122]. The bone microenvironment is rich in hematopoietic and bone cells, creating a fertile ground for tumor growth while simultaneously suppressing immune responses [75]. Clinical evidence supports the notion that patients with bone metastases experience reduced overall survival (OS) and progression-free survival (PFS) when treated with immunotherapy. A study by Brown et al. indicated that patients with advanced non-small-cell lung cancer (NSCLC) and bone metastases had a median OS of only 13.4 months compared to 18.8 months for those without bone involvement [63]. Similarly, highlighted that bone metastases are a negative prognostic factor, with randomized trials showing that patients with bone involvement had significantly shorter OS compared to those without metastases [123]. Furthermore, pointed out that the efficacy of novel therapies, including immunotherapy, on bone metastases is under-researched despite the high unmet medical need in this area [124]. The unique characteristics of the tumor microenvironment in bone metastases also play a crucial role in limiting the effectiveness of immune checkpoint inhibitors. It is crucial to report how the immune microenvironment in bone metastases differs from that in other metastatic sites, leading to variable responses to immunotherapy [73]. This is compounded by the fact that many clinical trials do not adequately stratify patients based on the site of metastasis, which may obscure the true efficacy of immunotherapeutic agents in this population [123]. Moreover, the combination of immunotherapy with other treatment modalities, such as radiotherapy, has shown promise in some cases, but the overall response rates remain low. For instance, while there are reports of synergistic effects when combining immunotherapy with palliative radiotherapy, the lack of consistent results underscores the complexity of treating bone metastases [125]. In conclusion, we can attribute the limited use of immunotherapy in patients with bone metastases to a combination of immunosuppressive factors within the bone microenvironment, reduced efficacy observed in clinical studies, and the unique challenges posed by the tumor microenvironment. Future research should focus on understanding these interactions better and developing targeted strategies to enhance the effectiveness of immunotherapy in this challenging patient population.

### 7.2. Preclinical Studies

#### 7.2.1. Mouse Model to Study Bone Metastases

Animal models are crucial for studying bone metastasis in cancer research, providing insights into the mechanisms of metastasis and aiding in the development of therapeutic strategies. The most commonly used animal models for studying bone metastasis include mouse models, which are favored for their ability to mimic human disease progression and response to treatments [126]. These models are complemented by innovative approaches such as 3D in vitro models, which address some limitations of traditional animal models.

Mouse models are extensively used to study bone metastases, a common site for tumor metastasis. These models utilize both human and murine tumor cell lines, such as prostate cancer PC-3 and breast cancer 4T1 or MDA-MB-231, to simulate the metastatic process. Techniques include circulatory system injection through the intracardiac approach, spine injection, intratibial injection, and spontaneous metastasis, each with specific applications and limitations [127,128]. Human-derived tumor cells are often inoculated into immunodeficient mice, such as athymic nude or NOD/SCID mice, to study bone metastasis. These models are crucial for understanding the pathophysiological mechanisms and testing new therapies, although they require stringent conditions and are prone to disease [129].

#### 7.2.2. Innovative Modeling Approaches

While mouse models remain the gold standard for studying bone metastasis, they have limitations such as high costs and low throughput. The development of 3D in vitro models and the integration of artificial intelligence for predictive modeling are promising advancements that could enhance the study of bone metastasis and accelerate drug development [130]. Magnetic Micro-Living-Motor (MLM) System: This novel approach enhances the precision of bone metastasis modeling by using a magnetic field to direct tumor cells to specific bone sites. It offers improved consistency and reduced metastasis to vital organs compared to traditional methods [129]. 3D In Vitro Models: These models aim to replicate the complex tumor microenvironment and bone remodeling processes. They are particularly useful for high-throughput drug screening and can mimic human cell interactions more accurately than animal models. For instance, a 3D model using breast cancer cell lines demonstrated the potential to study drug responses and bone resorption.

## 8. Checkpoint Inhibitors in Bone Metastases

The current understanding of the role of checkpoint inhibitors in preventing bone metastases in cancer patients is complex and evolving. While immune checkpoint inhibitors (ICIs) have revolutionized cancer treatment, several factors, including the immune microenvironment of the bone, the nature of the metastatic disease, and the inherent limitations of the current therapeutic approach, limit their efficacy in preventing bone metastases in cancer patients. Recent studies have explored various strategies to enhance the effectiveness of ICIs in this context, including combination therapies and targeting specific pathways within the bone microenvironment. As we advance in this field, collaboration between oncologists, immunologists, and researchers will be essential to translate these findings into clinical practice, ensuring that patients receive the most effective and individualized care possible. This multidisciplinary approach not only fosters innovation but also encourages the sharing of knowledge and resources, ultimately leading to breakthroughs that could transform the landscape of cancer treatment.

### Challenges in Treating Bone Metastases with ICIs

One of the primary challenges in treating bone metastases with ICIs is the immunosuppressive nature of the bone microenvironment. Studies have shown that bone metastases often exhibit unique immune profiles that differ significantly from those of primary tumors. For instance, the presence of myeloid-derived suppressor cells (MDSCs) and regulatory T cells (Tregs) in the bone can inhibit the anti-tumor immune response, thereby diminishing the effectiveness of ICIs [73]. Furthermore, the bone microenvironment can promote tumor cell survival and proliferation through various signaling pathways, such as the TGF-β pathway, which has been implicated in the progression of bone metastases and the development of resistance to immunotherapy (summarized in Pagnotti et al. [77]).

The efficacy of ICIs in patients with bone metastases is also hindered by the phenomenon of immune-related adverse events (irAEs). These adverse effects can manifest in the skeletal system, leading to complications such as osteonecrosis and pathological fractures, which can further complicate treatment [131,132]. The occurrence of these events may be indicative of an overly robust immune response triggered by ICIs, which, while beneficial in targeting cancer cells, can also lead to significant morbidity in patients with pre-existing bone lesions [131,132]. This duality of immune activation necessitates careful monitoring and management strategies to mitigate the risks associated with irAEs. The response evaluation criteria commonly used in clinical trials, such as the Response Evaluation Criteria in Solid Tumors (RECIST), may not adequately capture the unique response patterns seen in bone metastases treated with ICIs. Traditional imaging techniques may fail to distinguish between true tumor progression and inflammatory changes induced by the immune response, leading to potential misinterpretations of treatment efficacy. In a retrospective study, Li et al. [133] reported the impact of bone metastases (BoM) on clinical outcomes in patients with advanced non-small cell lung cancer (NSCLC) treated with immune checkpoint inhibitors (ICIs). It revealed that BoM significantly impairs the effectiveness of ICI monotherapy, with patients showing reduced progression-free survival (PFS) and overall survival (OS). However, the presence of BoM did not significantly affect outcomes when ICIs were combined with chemotherapy or anti-angiogenesis therapy, suggesting that these combined approaches might mitigate BoM’s adverse effects [133,134]. This limitation underscores the need for the development of more tailored assessment tools that can accurately reflect the therapeutic effects of ICIs in the context of bone metastases.

Another significant challenge is the heterogeneity of bone metastases themselves. Different types of cancers exhibit varying patterns of bone involvement, with some leading to osteolytic lesions while others result in osteoblastic changes. This heterogeneity can influence the immune landscape within the bone and thereby affect the response to ICIs [135,136]. For example, prostate cancer bone metastases often present with a blastic phenotype, which may be less responsive to ICIs compared to the lytic lesions seen in other cancers, such as breast cancer. Blastic metastases, characterized by excessive bone formation, showed enrichment in JAK-STAT pathway signaling and immune checkpoint markers such as PD-L1 and B7-H4, suggesting potential therapeutic targets. In contrast, lytic metastases, which lead to bone destruction, were associated with PI3K-AKT pathway activity and higher levels of immune cells, including macrophages and cytotoxic cells [135]. In a more recent study, prostate cancer can drive immunosuppression in bone metastases through a mechanism involving endothelial cell-to-osteoblast (EC-to-OSB) transition. This cancer-induced transition triggers the release of factors like Wnt ligands, CXCL14, and lysyl oxidase, which promote the recruitment and polarization of M2 macrophages. These macrophages suppress the proliferation and activity of CD8+ T cells, reducing the efficacy of immune checkpoint therapies [137]. Understanding these differences is crucial for optimizing treatment strategies and improving patient outcomes.

The timing of ICI administration in relation to other therapies, such as radiotherapy or systemic chemotherapy, can significantly affect treatment outcomes in patients with bone metastases. Combination therapies that integrate ICIs with other modalities may enhance therapeutic efficacy by modulating the immune microenvironment and overcoming resistance mechanisms [138]. However, the optimal sequencing and combination of these treatments remain to be fully elucidated, necessitating further research to establish evidence-based guidelines.

The identification of predictive biomarkers for response to ICIs in the context of bone metastases is another area of active investigation. Current research is actively investigating the molecular and immunological characteristics that may predict which patients are more likely to benefit from ICI therapy [139]. For instance, the expression levels of PD-L1 and other immune checkpoint molecules in the tumor microenvironment may serve as potential biomarkers for response, but their utility in the context of bone metastases is still being explored (reviewed by Xiang et al. [139]). The development of robust biomarkers could facilitate personalized treatment approaches and improve patient selection for ICI therapy. ICIs, such as nivolumab and pembrolizumab, have shown limited efficacy in treating bone metastases compared to other metastatic sites. This is attributed to the immunosuppressive nature of the bone microenvironment, which diminishes the responsiveness to ICIs [65,140].

The challenges associated with treating bone metastases using immune checkpoint inhibitors are multifaceted and require a comprehensive understanding of the unique interactions between the immune system and the bone microenvironment. Addressing these challenges will necessitate ongoing research efforts aimed at elucidating the underlying mechanisms of resistance, optimizing treatment strategies, and identifying predictive biomarkers. As the field of immunotherapy continues to evolve, it is imperative that clinicians remain vigilant in adapting their approaches to meet the specific needs of patients with bone metastases.

## 9. Combination Strategies

### 9.1. Combining Immunotherapy with Bone Targeted Agents (Bisphosphonates or Denosumab)

Combining ICIs with bone-targeted therapies, such as zoledronic acid or anti-angiogenic agents, has shown promise in improving treatment outcomes for bone metastases (Table 1). A retrospective study looked into the combined effects of denosumab, a drug that alters bone metabolism by inhibiting RANKL, along with immune checkpoint inhibitors (ICIs) for treating 29 patients who have been diagnosed with metastatic melanoma that has spread to the bones. The analysis showed remarkable treatment effectiveness, highlighted by an overall response rate (ORR) of 54% in the cohort administered a triplet therapy of nivolumab, ipilimumab, and denosumab, in contrast to a 50% ORR in those on dual treatment with PD-1 inhibitors and denosumab. Participants demonstrated radiographic enhancements in 62% of cases, as bone recalcification indicated. Significantly, the combinatorial therapeutic approach did not yield any unforeseen safety issues. The results suggest that the combination of denosumab and immune checkpoint inhibitors may provide a plausible and potentially impactful treatment method for patients encountering melanoma alongside bone metastases [141]. Another study looked at the clinical outcomes of combining denosumab with immune checkpoint inhibitors (ICIs) in patients with metastatic non-small cell lung cancer (NSCLC) who have skeletal metastases. The study retrospectively analyzed 69 patients, and the primary findings include that a longer overlap of denosumab and ICI therapy is associated with improved survival outcomes. Patients receiving more than three months of combined therapy had a median overall survival (OS) of 11.5 months compared to 3.6 months in those with less than three months of therapy. Progression-free survival (PFS) also improved with prolonged combination treatment [142]. A similar study was conducted to test the therapeutic effects of immune checkpoint inhibitors (ICIs), in combination with bone-targeted agents, on bone metastasis in advanced non-small-cell lung cancer (NSCLC). The study analyzed data from 29 patients treated with ICIs from 2016 to 2019, with a focus on the efficacy of ICIs in controlling both primary lung lesions and bone metastases. The most commonly used ICI was pembrolizumab, which showed significant therapeutic effects, especially when combined with denosumab. In 72.4% of the cases, the ICIs suppressed the progression of bone metastasis, with a complete response (CR) observed in 6.9% and a partial response (PR) observed in 17.2% of bone lesions [76]. A retrospective study was conducted to test the efficacy and Safety of concomitant immunotherapy and denosumab in patients with advanced non-small cell lung cancer with bone metastases aimed at evaluating the clinical outcomes of the combined treatment. The study found that while there were trends indicating better outcomes in the DI group, such as longer median progression-free survival (mPFS) and higher overall response rates (ORRs), these differences were not statistically significant across the entire cohort. However, in patients without certain genetic mutations (the non-driver cohort), the combination therapy showed a significant improvement in mPFS compared to control groups. This study also confirmed that the combination of denosumab with ICIs did not lead to a significant increase in adverse events, making it a relatively safe treatment option [143].

In contrast to the results from these studies, patients with metastatic non-small cell lung cancer (mNSCLC) treated with immune checkpoint inhibitors (ICIs) were found to have significantly shorter overall survival in patients with bone metastases compared to patients without bone metastases, even after controlling for various clinical factors. The median overall survival was 5.9 months for patients with bone metastases versus 13.4 months for those without. The use of bone-modifying agents (BMAs) was not associated with reduced SREs or improved survival [74]. Future studies are recommended to validate these findings and to further explore the mechanisms behind the contradicting effects of ICIs and bone-modifying agents. A multidisciplinary approach, including systemic treatment with ICIs and BMAs, may be beneficial in managing advanced NSCLC with bone metastasis.

**Figure 2:**
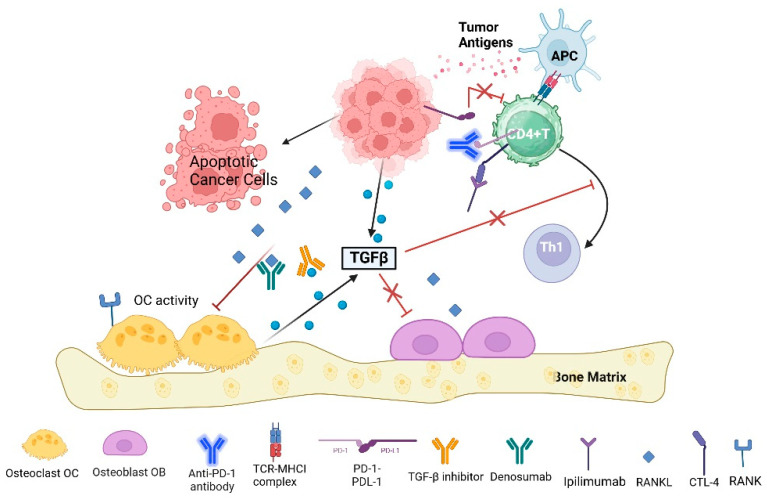
Modulation of Bone Metastasis by Immune Checkpoint and Signaling Inhibitors. This figure depicts the intricate interplay between the immune system and bone remodeling mechanisms in the context of bone metastases. Key elements include the immune checkpoints (PD-1/PD-L1 axis) and T cell receptor (TCR-MHCI complex) interactions that play crucial roles in immune evasion by tumor cells. The figure also highlights the impact of therapeutic agents such as anti-PD-1 antibodies, TGFβ inhibitors, and bone-targeted agents like Denosumab on these pathways. These treatments not only target immune suppression but also interfere with the osteoclastogenic activity mediated by RANKL, pivotal in bone resorption. Additionally, the use of CTLA-4 inhibitors like Ipilimumab further illustrates the therapeutic strategies to enhance anti-tumor immunity. This representation serves as a comprehensive guide to understanding the dual role of the immune system in promoting and inhibiting bone metastasis (Figure was created with BioRender.com).

The enhanced efficacy of combining bisphosphonates, specifically nitrogen-containing bisphosphonates (N-BPs), with immunotherapy in cancer treatment can be attributed to several underlying mechanisms (Figure 2). N-BPs are known to activate Vγ9Vδ2 T lymphocytes, which play a crucial role in the immune response against tumors by producing inflammatory cytokines and exhibiting strong cytolytic activities [150]. This activation enhances the anti-tumor effector functions of these γδ T cells, making them more effective in targeting malignancies. Moreover, the immunomodulatory properties of bisphosphonates may contribute to their antineoplastic effects, providing a synergistic mechanism when used alongside immunotherapy. The γδ T-cell receptor on these T cells allows for the intrinsic recognition of malignancy-associated antigens, further boosting the anti-tumor response. Besides their direct effects on γδ T cells, bisphosphonates may also enhance the therapeutic efficacy of existing cancer treatments through their ability to modulate the tumor microenvironment. By inhibiting osteoclastic activity and altering bone remodeling processes, these compounds can reduce the availability of growth factors that tumors exploit for survival [151]. Thus, the combination of bisphosphonates with immunotherapy not only enhances the activation of immune cells but also leverages their ability to recognize and respond to cancer cells, potentially leading to improved treatment outcomes in cancer patients.

While the results indicate a potential benefit from the concurrent use of ICIs and denosumab, there is a need for prospective clinical trials to confirm these findings and explore the mechanisms of synergy between RANKL inhibitors and immunotherapies. For example, DKK1 blockade combined with zoledronic acid demonstrated synergistic effects in controlling bone metastases. Similarly, the combination of ICIs with anti-angiogenic agents improved progression-free survival in NSCLC patients with bone metastases [152]. There is a critical need for more comprehensive studies to understand the interplay between immune cells, ICIs, and the bone-tumor microenvironment. This includes exploring novel therapeutic targets and strategies to enhance the efficacy of ICIs in bone metastases [140,153].

Despite the fact that ICIs have transformed cancer treatment, their role in preventing bone metastases remains limited due to the unique challenges posed by the bone microenvironment.

### 9.2. Combining with Chemotherapy: Potential Synergies

The treatment of bone metastases, particularly through the combination of immunotherapy and chemotherapy, has emerged as a promising strategy in oncology. This approach seeks to exploit the potential synergies between these two modalities, enhancing therapeutic efficacy while potentially mitigating the adverse effects commonly associated with each treatment type (Table 1). The rationale for combining immunotherapy with chemotherapy lies in their complementary mechanisms of action, which can lead to improved patient outcomes in various malignancies, including those with bone metastases.

Bone metastases are often associated with significant morbidity and poor prognosis, particularly in cancers such as breast, prostate, and lung cancer. The presence of metastatic disease in the bone can alter the tumor microenvironment, creating an immunosuppressive milieu that complicates treatment efforts. For instance, the bone marrow is rich in immunosuppressive cells and factors that can hinder the effectiveness of immunotherapy [154,155]. Therefore, strategies that combine immunotherapy with chemotherapy may help to overcome these barriers by enhancing immune activation while simultaneously targeting tumor cells directly.

Recent studies have shown that the combination of chemotherapy and immunotherapy can lead to synergistic effects. For example, Liu et al. reported a case of significant reossification in a patient with metastatic urothelial carcinoma treated with pembrolizumab, suggesting that immunotherapy can positively influence bone health in metastasis [156]. Similarly, Kuo and Lin highlighted the successful regression of advanced maxillary sinus cancer with orbital invasion through a regimen combining chemotherapy and immunotherapy, underscoring the potential of this combined approach in treating complex metastatic disease [157]. Moreover, the combination of chemotherapy with immunotherapy has been shown to enhance anti-tumor efficacy in preclinical models. Huang et al. demonstrated that intravesical xenogeneic urothelial cell immunotherapy combined with chemotherapy significantly improved outcomes in murine bladder tumor models, indicating that such combinations can effectively stimulate immune responses while also directly targeting tumor cells [144]. This is particularly relevant in the context of bone metastases, where the immune response can be further bolstered by the cytotoxic effects of chemotherapy.

The timing and sequencing of chemotherapy and immunotherapy administration are critical factors influencing treatment outcomes. Studies suggest that administering chemotherapy prior to immunotherapy may enhance the immune response by increasing the release of tumor antigens and promoting T-cell activation [145]. For instance, studies have shown that administering chemotherapy before immunotherapy enhances the efficacy of treatment in various cancer types, including esophageal squamous cell carcinoma [145]. This sequential approach may be particularly beneficial in patients with bone metastases, where the immune landscape is often altered. Furthermore, the immune-modulating effects of certain chemotherapeutic agents can enhance the efficacy of subsequent immunotherapy. For example, chemotherapies like cabozantinib have been noted for their immune-priming effects, which can sensitize tumors to immunotherapeutic agents [158]. This synergy is particularly important in the treatment of bone metastases, where the tumor microenvironment can be resistant to conventional therapies.

In addition to enhancing immune responses, the combination of chemotherapy and immunotherapy can also mitigate the adverse effects associated with each treatment modality. For instance, immunotherapy has been associated with fewer hematological toxicities compared to chemotherapy, making it a valuable adjunct in patients who may be vulnerable to the side effects of traditional cytotoxic agents [159]. This is particularly relevant in the context of bone metastases, where maintaining bone marrow function is crucial for overall patient health.

The potential for combination therapies is further supported by the findings of Vanneman and Dranoff, who emphasized the importance of integrating immunotherapy with other treatment modalities to maximize clinical benefits [160]. Their review highlighted various mechanisms through which chemotherapy can enhance the efficacy of immunotherapy, including modulation of the tumor microenvironment and enhancement of antigen presentation.

The integration of novel drug delivery systems, such as nanoparticles, into the treatment paradigm of bone metastases also holds promise. Nanoparticle-mediated drug delivery can enhance the targeting of both chemotherapeutic and immunotherapeutic agents to the tumor site, potentially improving therapeutic outcomes while minimizing systemic toxicity [161]. This innovative approach could be particularly beneficial in the treatment of bone metastases, where localized delivery may enhance drug efficacy.

The combination of immunotherapy and chemotherapy presents a multifaceted approach to treating bone metastases, leveraging the strengths of both modalities to improve patient outcomes. The synergistic effects observed in preclinical and clinical studies underscore the potential of this strategy to enhance anti-tumor immunity while directly targeting metastatic lesions.

### 9.3. Radiation and Immunotherapy: Abscopal Effect

The combined effects of radiation therapy and immunotherapy in the treatment of bone metastases represent a significant advancement in oncology, particularly due to the potential for enhanced therapeutic efficacy and the induction of the abscopal effect. The abscopal effect refers to the phenomenon where localized radiation treatment not only affects the irradiated tumor but also induces systemic antitumor responses in distant, non-irradiated tumors. The release of tumor-associated antigens following radiation-induced cell death is believed to activate the immune system, mediating this effect [146,147,148].

Radiation therapy has long been used as a primary treatment modality for localized tumors, but its role has evolved with the advent of immunotherapy (Table 1). Studies have demonstrated that the combination of these two modalities boosts the immune response to tumors, particularly in cases of metastatic disease. For instance, studies have demonstrated that radiation can stimulate the immune system by increasing the expression of immune checkpoints and enhancing the visibility of tumor antigens, thereby promoting a systemic immune response that can target both irradiated and non-irradiated tumors [162,163,164,165]. This synergistic effect is particularly relevant in the context of bone metastases, where traditional treatment options may be limited, and the potential for systemic disease progression is high.

Radiation therapy can induce immunogenic cell death, characterized by the release of damage-associated molecular patterns (DAMPs) and tumor-associated antigens, which can prime the immune system to recognize and attack metastatic lesions [166,167,168]. The combination of radiation with immune checkpoint inhibitors, such as anti-PD-1 or anti-CTLA-4 therapies, has been shown to enhance the abscopal response by overcoming the immunosuppressive tumor microenvironment often present in metastatic disease [169,170,171,172]. This combination approach not only improves local control of the irradiated tumor but also has the potential to extend the benefits to distant metastatic sites.

Clinical evidence supporting the efficacy of combined radiation and immunotherapy is growing. For example, several case studies and clinical trials have reported instances of abscopal responses in patients with metastatic melanoma and renal cell carcinoma following treatment with radiation and immune checkpoint inhibitors [173,174,175,176]. Administering radiation in conjunction with immunotherapy often leads to optimal outcomes, highlighting the significance of timing and sequencing in these therapies [177,178,179].

Moreover, the role of the immune system in mediating the abscopal effect has been further elucidated through preclinical studies. Research has shown that radiation can enhance the activation and proliferation of T cells, which are crucial for eliciting a robust immune response against tumors [180,181]. The induction of a favorable immune environment through radiation not only promotes local tumor control but also facilitates the recognition and elimination of distant metastases, thereby improving overall survival rates in patients with advanced cancer [182,183,184].

Despite the promising results, challenges remain in optimizing the combination of radiation and immunotherapy. Factors such as tumor heterogeneity, immune-suppressive cells in the tumor microenvironment, and the timing of treatment can significantly influence the efficacy of this combined approach [185,186]. Ongoing clinical trials are essential to address these challenges and to refine treatment protocols that maximize the therapeutic benefits while minimizing potential toxicities [187,188]. A retrospective study was conducted on patients with melanoma and bone metastases. The study analyzed 305 patients from 19 Italian centers, focusing on factors like skeletal-related events (SREs) and survival outcomes. It found that 83% of patients had metachronous bone metastases, primarily affecting the spine, and 47% experienced at least one SRE, with palliative radiotherapy being the most common treatment. The study revealed that while no melanoma-specific factors predicted SREs, the early use of bone-targeting agents significantly reduced the risk and delayed the occurrence of SREs. Median overall survival (OS) was 10.7 months, but patients who received both immunotherapy and radiotherapy had the best outcomes, with a median OS of 16.5 months, suggesting a potential synergistic effect between immunotherapy and radiotherapy [149].

Integrating radiation therapy and immunotherapy represents a paradigm shift in the management of bone metastases and other advanced cancers. The ability of radiation to induce systemic immune responses, exemplified by the abscopal effect, offers a compelling rationale for this combination strategy.

### 9.4. Combining Checkpoint Inhibitors with Other Immunotherapies

The treatment of bone metastases, particularly through the combination of immune checkpoint inhibitors (ICIs) with other immunotherapies, has emerged as a promising area of research in oncology (Table 1). Bone metastases present unique challenges due to their complex microenvironment, which can suppress immune responses and promote tumor progression. Recent studies have elucidated the potential benefits of combining ICIs with various therapeutic modalities to enhance anti-tumor immunity and improve clinical outcomes in patients with bone metastases.

One of the primary mechanisms by which ICIs exert their effects is by reactivating T cells that have been rendered inactive by the tumor microenvironment. For instance, Arellano et al. demonstrated that T cells suppressed by the bone microenvironment contribute to increased osteoclast formation and osteolytic bone metastases in murine models, showing a need for therapies that can counteract this suppression [189]. The combination of ICIs with agents that can modulate the immune microenvironment, such as trabectedin, has shown promise in preclinical settings. Ratti et al. reported that trabectedin can recruit T cells to the tumor site, potentially converting “cold” tumors into “hot” ones and enhancing the efficacy of ICIs [190].

The bone microenvironment is known to harbor various immune cells, including myeloid-derived suppressor cells (MDSCs) and regulatory T cells (Tregs), which can inhibit effective anti-tumor responses [191]. The resistance to conventional therapies can be attributed to the interplay between these cells and tumor cells. Thus, combining ICIs with treatments that target these immune suppressive pathways may yield better outcomes. For example, the use of bisphosphonates in conjunction with ICIs has been suggested to enhance the therapeutic efficacy in patients with non-small cell lung cancer (NSCLC) and bone metastases [133].

Clinical evidence supports the notion that combination therapies can lead to improved survival rates and quality of life for patients with bone metastases. A study by Qiang et al. highlighted that pembrolizumab when used in combination with other therapies such as radiotherapy and bone-targeted agents, showed a synergistic effect in managing bone metastases in advanced NSCLC [125]. He reported that patients receiving first-line pembrolizumab exhibited better outcomes in terms of objective response rate (ORR), progression-free survival (PFS), and overall survival (OS) compared to those who received it as a second-line or later treatment. This aligns with findings from other studies that show the potential for ICIs to alleviate pain and improve overall survival when combined with palliative treatments such as radiotherapy [74].

Liu et al. emphasized that the specific microenvironment of bone metastases poses challenges for ICI therapy, as it can hinder immune activation and response [73]. Therefore, strategies that aim to modify this microenvironment, such as the use of CAR T-cell therapies that are engineered to target bone metastases, have been proposed [192]. These approaches may enhance the recruitment and activation of T cells within the bone, potentially leading to better therapeutic outcomes. Integrating novel immunotherapeutic strategies, such as the use of immune checkpoint inhibitors alongside targeted therapies, has also shown promise. For instance, the combination of pembrolizumab with axitinib has been explored in renal cell carcinoma, demonstrating improved response rates in patients with bone metastases [193]. This suggests that the synergistic effects of combining ICIs with targeted agents can enhance the overall efficacy of treatment regimens for patients suffering from metastatic disease. In addition to these combinations, the use of local therapies, such as radiotherapy in conjunction with ICIs, has been investigated. The abscopal effect, where localized radiation leads to systemic anti-tumor responses, has been observed in patients receiving concurrent immunotherapy [194]. This phenomenon underscores the potential for combining local and systemic therapies to enhance treatment efficacy in bone metastases.

Despite the promising results from various studies, the efficacy of ICIs in patients with bone metastases remains an area of active investigation. Some studies have associated the presence of bone metastases with poorer outcomes, indicating the need for further research to fully understand the implications of bone involvement in treatment responses [133]. The development of predictive models that account for the unique characteristics of bone metastases could help tailor immunotherapy approaches to individual patients, thereby improving clinical outcomes.

The combination of immune checkpoint inhibitors with other immunotherapies presents a promising avenue for the treatment of bone metastases. The unique challenges posed by the bone microenvironment necessitate innovative strategies that can enhance immune responses and improve patient outcomes.

## 10. Challenges and Limitations

### 10.1. Immune Escape Mechanisms Employed by Tumor Cells in Bone Metastases

One of the primary mechanisms of immune escape in bone metastases is the alteration of the immune microenvironment itself. Tumor cells can manipulate the bone microenvironment to create an immunosuppressive niche that inhibits effective immune responses. For instance, the presence of myeloid-derived suppressor cells (MDSCs) in the bone marrow has been shown to suppress T cell activation and promote tumor progression [8,189]. These MDSCs can inhibit the proliferation and function of cytotoxic T lymphocytes (CTLs), thereby allowing tumor cells to evade immune detection [195]. Additionally, the bone marrow is rich in regulatory T cells (Tregs) and other immunosuppressive factors that further contribute to this immune evasion [196]. The expression of immune checkpoint molecules such as PD-L1 is another critical aspect of immune escape in bone metastases. Tumor cells can upregulate PD-L1 expression in response to immune pressure, which interacts with PD-1 on T cells to inhibit their activity [73]. This interaction is particularly significant in the bone microenvironment, where the presence of immune checkpoints can lead to T cell exhaustion and reduced anti-tumor immunity [74]. The secretion of immunosuppressive cytokines such as TGFβ by tumor cells not only promotes tumor growth but also suppresses the immune response, facilitating immune escape [77].

The role of tumor-associated macrophages (TAMs) in bone metastases is also significant. TAMs can adopt a pro-tumorigenic phenotype that supports tumor growth and metastasis while simultaneously suppressing anti-tumor immunity [83]. These macrophages can secrete various cytokines and growth factors that promote tumor cell survival and proliferation, creating a feedback loop that enhances immune evasion. Moreover, the interaction between TAMs and tumor cells can lead to the secretion of factors that further inhibit T-cell function and promote an immunosuppressive environment [83].

Additionally, the phenomenon of immune editing plays a significant role in the immune escape of tumor cells in bone metastases. Tumor cells can undergo genetic and epigenetic changes that allow them to lose immunogenicity, making them less recognizable to the immune system [71]. This process can involve the downregulation of major histocompatibility complex (MHC) molecules, which are crucial for T cell recognition of tumor antigens [197]. By reducing the expression of these molecules, tumor cells can effectively hide from immune surveillance, allowing them to persist and proliferate within the bone microenvironment. The bone microenvironment itself can influence the immune response. Tumor cells can alter the presence of osteoclasts and osteoblasts, which are critical for bone remodeling to create a supportive niche for metastasis [196]. Tumor cells can secrete factors that promote osteoclastogenesis, leading to bone resorption and the release of growth factors that further support tumor growth [198]. This dynamic interaction not only facilitates tumor growth but also contributes to the immunosuppressive nature of the bone microenvironment.

### 10.2. Immune-Related Adverse Effects

One of the primary immune-related adverse events associated with immunotherapy in patients with bone metastases is the exacerbation of pre-existing conditions or the emergence of new autoimmune phenomena. For instance, the immunosuppressive microenvironment created by bone metastases can lead to reduced efficacy of immune checkpoint inhibitors (ICIs) and may provoke irAEs (Table 2), such as pneumonitis, colitis, and endocrinopathies [124]. The presence of bone metastases has been shown to correlate with poorer clinical outcomes in patients receiving ICIs, suggesting that the bone microenvironment may not only hinder the effectiveness of immunotherapy but also contribute to the development of irAEs [124].

The bone marrow niche, which is rich in immunosuppressive cells and factors such as transforming growth factor-beta (TGFβ), plays a significant role in this context. TGFβ is known to inhibit T cell activation and proliferation, thereby contributing to immune evasion by tumor cells in the bone [120]. This immunosuppressive environment can lead to a paradoxical increase in autoimmune reactions as the immune system attempts to overcome the local suppression, resulting in irAEs that can complicate the clinical management of these patients [73,122]. The management of irAEs in patients with bone metastases requires careful consideration of the underlying bone pathology. For example, the use of corticosteroids to manage severe irAEs can exacerbate bone health issues, such as osteoporosis and the risk of pathological fractures [133,199]. Therefore, a multidisciplinary approach that includes oncologists, endocrinologists, and pain management specialists is essential for effectively managing these adverse events while minimizing the impact on bone health [200,201].

In addition to corticosteroids, other management strategies for irAEs may involve the use of immunosuppressive agents, such as infliximab for colitis or mycophenolate mofetil for severe dermatologic reactions [124,133]. However, it is important to consider the potential for further immunosuppression, which could worsen the underlying cancer or result in additional complications, when using these agents [124]. The timing and dosage of these interventions are critical, as delayed management of irAEs can lead to more severe outcomes and prolonged hospitalizations [122,202].

Another significant aspect of managing immunotherapy-related toxicities in patients with bone metastases is the role of bone-modifying agents (BMAs), such as bisphosphonates and denosumab. These agents are commonly used to prevent skeletal-related events (SREs) in patients with bone metastases, but their interaction with immunotherapy can complicate treatment regimens [199,201]. For instance, denosumab has been associated with hypocalcemia, which can be exacerbated by the immunotherapy-induced inflammatory response [202,203]. Therefore, careful monitoring of calcium levels and proactive management of hypocalcemia are essential components of care for these patients [199,202].

Integrating supportive care measures, including pain management and physical rehabilitation, is also vital in the comprehensive management of patients receiving immunotherapy for bone metastases. Effective pain control can improve patient quality of life and adherence to immunotherapy regimens [199,204]. Physical therapy can help mitigate the functional decline associated with both the cancer and its treatment, thereby enhancing overall patient outcomes [199,204].

## 11. Future Directions

Future directions in utilizing immunotherapy for bone metastases within personalized medicine are promising, particularly through the exploration of adoptive immunotherapy and the role of specific immune cell subsets. One potential avenue is the use of antigen-specific T lymphocytes, which have shown early promise in targeting skeletal metastases, although this approach has yet to be fully explored in clinical settings [205]. The activation of effector T lymphocytes, particularly CD8+ cells, has demonstrated significant tumor regression in preclinical models, indicating their critical role in effective immunotherapeutic strategies. Moreover, integrating NK cell-based therapies could enhance treatment efficacy, as targeting molecular ligands such as NKG2D and DNAM-1 may improve the immune response against bone metastases. As research progresses, combining these immunotherapeutic strategies with traditional treatments could lead to more effective personalized medicine approaches for patients suffering from bone metastases.

One of the key areas of focus is the identification and targeting of specific biomarkers that can predict the response to immunotherapy. Recent studies have highlighted the potential of various biomarkers, including circulating tumor cells (CTCs), micro-RNAs, and proteins such as osteoprotegerin (OPG) and insulin-like growth factors (IGFs), which are involved in the pathogenesis of bone metastases [191,206,207]. For instance, the identification of gene expression profiles associated with bone metastases can help in stratifying patients based on their likelihood of benefiting from immunotherapeutic agents [208,209]. This personalized approach not only enhances the precision of treatment but also aids in the early detection of metastases, which is crucial for effective intervention [210].

In addition to biomarker identification and immune modulation, the development of novel therapeutic agents that specifically target the bone microenvironment is gaining traction. Recent advancements in drug delivery systems, such as bisphosphonates and radiopharmaceuticals, have shown promise in selectively delivering therapeutic agents to bone metastases [211,212]. These agents can be combined with immunotherapeutic strategies to enhance local immune responses while minimizing systemic toxicity. For instance, the use of radioactive platinum-bisphosphonate compounds has been explored for their potential to deliver targeted therapy to areas of high metabolic activity in bone metastases [211].

In conclusion, a comprehensive approach that integrates biomarker identification, immune modulation, targeted therapies, combination treatments, and supportive care strategies characterizes the future directions in utilizing immunotherapy for bone metastases within personalized medicine. As research continues to evolve, the potential to tailor immunotherapeutic interventions to the unique characteristics of each patient’s cancer will likely lead to improved outcomes and a better quality of life for those affected by bone metastases.

## Figures and Tables

**Table 1:** Comparative Summary of Combination Therapies in Bone Metastases.

Combination Therapy	Therapeutic Agents	Mechanisms of Action	Clinical Outcomes	Key Studies & Findings	Challenges and Limitations
ICIs + Bone-Targeted Agents	Denosumab, Zoledronic Acid	ICIs restore T-cell activity by blocking immune checkpoint pathways (PD-1/PD-L1 or CTLA-4); denosumab inhibits RANKL-mediated osteoclastogenesis [8,141,142].	Prolonged progression-free survival in NSCLC patients; improved outcomes in melanoma with bone metastases [8,141].	Combination of denosumab and nivolumab showed significant overall response rates in patients with metastatic melanoma [141].	Immunosuppressive bone microenvironment; limited stratification of clinical trial patients by metastasis sites; inconsistent benefits in different cancers [8,142].
ICIs + Chemotherapy	Pembrolizumab + Cytotoxic Agents	Chemotherapy induces immunogenic cell death and tumor antigen release, enhancing ICI efficacy [144,145].	Tumor regression and reossification observed in bone metastases [144].	Enhanced T-cell activation observed when chemotherapy precedes ICI therapy [145].	Lack of optimized sequencing and dosing; potential overlapping toxicities [8,144].
ICIs + Radiotherapy	Nivolumab + Local Radiotherapy	Radiation induces immunogenic cell death, releasing antigens that stimulate systemic immune responses and the abscopal effect [8,146,147,148].	Improved survival rates and systemic responses in metastatic cancers, including melanoma [146,149].	Timing and sequencing critical; abscopal effect observed in some cases but inconsistent outcomes in others [148,149].	Heterogeneity in tumor microenvironment and immune suppression; variable effectiveness across tumor types [8,148].
CAR T-cell Therapy + ICIs	Genetically Modified T-cells	CAR T-cells target tumor antigens directly; ICIs prevent immune exhaustion by blocking inhibitory checkpoints [8,98,101].	Effective in hematologic malignancies; limited efficacy in solid tumors like bone metastases [101].	Potential synergy with checkpoint inhibitors demonstrated in preclinical studies [101].	Immunosuppressive bone microenvironment; high risk of cytokine release syndrome; challenges in solid tumors [98,104].
Bone-Targeted Agents + Chemotherapy	Bisphosphonates + Cytotoxic Agents	Bisphosphonates reduce osteoclast activity and bone resorption; chemotherapy induces cytotoxicity and immune modulation [150,151].	Stabilization of bone lesions noted; impact on overall survival remains unclear [8,151].	Synergy in reducing bone resorption and enhancing tumor antigen presentation [150].	Long-term outcomes unclear; risk of combined toxicities; limited data on efficacy in bone metastases [8,150].
ICIs + Anti-Angiogenic Agents	Nivolumab + Bevacizumab	Anti-angiogenics normalize tumor vasculature, enhancing ICI delivery and immune cell infiltration into the tumor microenvironment [8,152].	Improved progression-free survival in NSCLC patients with bone metastases [152].	Evidence supports synergistic effects of ICIs and anti-angiogenic agents in reducing tumor progression [152].	Risk of immune-related adverse events (irAEs); increased toxicity; need for robust predictive biomarkers [8,152].

This table provides an overview of combination therapeutic strategies targeting bone metastases, focusing on immune checkpoint inhibitors (ICIs) combined with various agents. The table compares therapeutic agents, their mechanisms of action, observed clinical outcomes, supporting key studies, and the challenges or limitations associated with each approach.

**Table 2:** Comparative Overview of Side Effects in Combination Therapies for Bone Metastases.

Combination Therapy	Common Side Effects	Severe Side Effects
ICIs + Bone-Targeted Agents	Fatigue, musculoskeletal pain, and mild skin rash associated with ICIs and bone agents like denosumab [141,142].	Osteonecrosis of the jaw (rare, linked to bone-targeted agents); immune-related adverse events (irAEs) such as pneumonitis [142].
ICIs + Chemotherapy	Nausea, diarrhea, and mild hematologic toxicity such as anemia [144,145].	Severe neutropenia, febrile infections, and exacerbation of chemotherapy-induced toxicities [145]
ICIs + Radiotherapy	Fatigue and localized skin irritation from radiotherapy; flu-like symptoms from ICIs [8,148].	Severe irAEs, including pneumonitis, colitis, or inflammatory changes misinterpreted as disease progression [8,149]
CAR T-cell Therapy + ICIs	Fever, fatigue, and mild cytokine release syndrome (CRS) symptoms like hypotension and tachycardia [98,104].	Severe CRS, neurotoxicity, and life-threatening immune hyperactivation (e.g., macrophage activation syndrome) [104,105].
Bone-Targeted Agents + Chemotherapy	Fatigue, mild nausea, and increased bone pain during initial treatment (flare reaction) [150,151].	Long-term osteonecrosis of the jaw; severe myelosuppression or cumulative toxicities with chemotherapy [151]
ICIs + Anti-Angiogenic Agents	Hypertension, proteinuria, and fatigue due to anti-angiogenic agents like bevacizumab; mild irAEs from ICIs [152].	Severe thromboembolic events, gastrointestinal perforation, or exacerbated irAEs (e.g., colitis or hepatitis) [152]

This table summarizes the side effect profiles of combination therapies used in the treatment of bone metastases, categorizing them into Common Side Effects and Severe Side Effects. The therapies evaluated include immune checkpoint inhibitors (ICIs) combined with agents such as bone-targeted treatments, chemotherapy, radiotherapy, CAR T-cell therapy, and anti-angiogenic agents.
